# Predicting the Outcome of Patients with Aneurysmal Subarachnoid Hemorrhage: A Machine-Learning-Guided Scorecard

**DOI:** 10.3390/jcm12227040

**Published:** 2023-11-10

**Authors:** Yi Zhang, Hanhai Zeng, Hang Zhou, Jingbo Li, Tingting Wang, Yinghan Guo, Lingxin Cai, Junwen Hu, Xiaotong Zhang, Gao Chen

**Affiliations:** 1 Department of Neurosurgery, Second Affiliated Hospital of Zhejiang University School of Medicine, Zhejiang University, Hangzhou 310016, China; 2 Key Laboratory of Precise Treatment and Clinical Translational Research of Neurological Diseases, Hangzhou 310016, China; 3 Department of Neurointensive Care Unit, Second Affiliated Hospital of Zhejiang University School of Medicine, Zhejiang University, Hangzhou 310016, China; 4 College of Electrical Engineering, Zhejiang University, Hangzhou 310020, China; 5 Interdisciplinary Institute of Neuroscience and Technology, College of Biomedical Engineering & Instrument Science, Zhejiang University, Hangzhou 310020, China; 6 MOE Frontier Science Center for Brain Science and Brain-Machine Integration, Zhejiang University, Hangzhou 310058, China

**Keywords:** aneurysmal subarachnoid, hemorrhage, machine learning, prediction, scorecard

## Abstract

Aneurysmal subarachnoid hemorrhage (aSAH) frequently causes long-term disability, but predicting outcomes remains challenging. Routine parameters such as demographics, admission status, CT findings, and blood tests can be used to predict aSAH outcomes. The aim of this study was to compare the performance of traditional logistic regression with several machine learning algorithms using readily available indicators and to generate a practical prognostic scorecard based on machine learning. Eighteen routinely available indicators were collected as outcome predictors for individuals with aSAH. Logistic regression (LR), random forest (RF), support vector machines (SVMs), and fully connected neural networks (FCNNs) were compared. A scorecard system was established based on predictor weights. The results show that machine learning models and a scorecard achieved 0.75~0.8 area under the curve (AUC) predicting aSAH outcomes (LR 0.739, RF 0.749, SVM 0.762~0.793, scorecard 0.794). FCNNs performed best (~0.95) but lacked interpretability. The scorecard model used only five factors, generating a clinically useful tool with a total cutoff score of ≥5, indicating poor prognosis. We developed and validated machine learning models proven to predict outcomes more accurately in individuals with aSAH. The parameters found to be the most strongly predictive of outcomes were NLR, lymphocyte count, monocyte count, hypertension status, and SEBES. The scorecard system provides a simplified means of applying predictive analytics at the bedside using a few key indicators.

## 1. Introduction

Aneurysmal subarachnoid hemorrhage (aSAH) is an acute and devastating central nervous system disease, affecting approximately one in ten thousand individuals annually [1]. Despite appropriate treatment, approximately 20% of survivors continue to experience impaired functioning following discharge due to neurological deficits [2]. Predicting outcomes in patients with aSAH is, therefore, meaningful for prognosis and guiding individualized treatment decisions on admission.

Traditional prognostic models rely on statistical techniques like univariate and multivariate logistic regression analysis [3,4,5]. Machine learning employs algorithms, enabling computers to learn and function without explicit programming. Predictions using machine-learning-assisted algorithms have shown superior performance [6,7,8]. Both approaches typically incorporate demographic variables (e.g., age, hypertension), clinical grading scales (e.g., Hunt and Hess, World Federation of Neurological Surgeons (WFNS) grade, Glasgow Coma Scale), and neuroimaging assessments (Fisher grade) as predictors.

Recently, some laboratory indices have also contributed to outcome prediction. For example, C-reactive protein (CRP), produced by the liver, increases during inflammation and independently predicts outcomes in the SAHIT model [9]. The neutrophil-to-lymphocyte ratio (NLR), an accessible inflammatory marker, also aids prognosis [10,11]. Cerebrospinal fluid (CSF) metabolites, like vasoactive molecules, predict poor outcomes in aSAH patients [12].

Machine learning is transforming domains through automatic, adaptive, nonlinear, scalable, and flexible learning. Recent natural language processing advances like ChatGPT showcase progress in transformer models and unsupervised learning. However, limitations like poor interpretability persist. Moreover, complex algorithms remain impractical for most clinicians. Therefore, this study primarily aims to evaluate logistic regression and machine learning methods using readily available data, including demographics, clinical grading scales, CT metrics (e.g., Subarachnoid Hemorrhage Early Brain Edema Score (SEBES) [13] and Barrow Neurological Institute (BNI) scale [14]), and routine blood tests to predict aSAH outcomes. Furthermore, we will generate a simplified prognostic scorecard from highly predictive machine learning factors for clinical utility.

## 2. Materials and Methods

### 2.1. Study Population

This was a retrospective cohort study conducted at the Department of Neurosurgery, Second Affiliated Hospital of Zhejiang University. The study protocol was approved by the Human Ethics Committee of the Second Affiliated Hospital of Zhejiang University in accordance with the ethical guidelines of the Helsinki Declaration.

The model development cohort included consecutive patients admitted between May 2018 and July 2020 who were older than 18 years and had CT angiography (CTA) or digital subtraction angiography (DSA) and initial blood testing within 24 h of aneurysmal subarachnoid hemorrhage (SAH) symptom onset. Patients with hematological diseases, infections, and other systemic conditions (e.g., severe renal/hepatic dysfunction, malignant neoplasms) were excluded. A separate cohort, meeting identical inclusion/exclusion criteria and admitted between March 2021 and August 2022, was used for external validation of the risk score model. Written informed consent was obtained from participants or their legal guardians.

Eighteen candidate predictors were collected: (1) age; (2) gender; (3) aneurysm location (anterior cerebral artery, middle cerebral artery, posterior circulation, or internal carotid artery); (4) chronic hypertension; (5) treatment (coil, clip, or conservative treatment); (6) GCS score; (7) WFNS grade; (8) Hunt and Hess scale; (9) mFisher grade; (10) SEBES; (11) BNI scale; (12) intraventricular hemorrhage; (13) neutrophil count; (14) lymphocyte count; (15) monocyte/macrophage count; (16) eosinophil count; (17) basophil count; (18) NLR (absolute neutrophil count divided by absolute lymphocyte count).

The primary outcome was 3-month functional status measured by the modified Rankin Scale (mRS). Poor outcome was defined as mRS ≥ 3. Outcome assessment was performed by a trained nurse through telephone follow-up and confirmed via chart review by a neurosurgeon blinded to the prediction model scores.

### 2.2. Statistics

Continuous variables were expressed as mean ± standard deviation (SD) or median with interquartile range (IQR). The aforementioned statistical analysis was conducted using “pandas” [15] and “Scikit-learn” package (Version 0.24.2) [16] in Python. The importance of predictor factors in each model was scaled through max-min scaling and visualized using heatmaps created with the “pheatmap” package (Version 1.0.12) [17] in R.

### 2.3. Model Analysis

Logistic regression, random forest, support vector machines (SVMs), and fully connected neural networks (FCNNs) were utilized in this study. Firstly, in the binary regression model, the variance inflation factor (VIF) was applied to eliminate multicollinearity and ensure the least square method (removing predictors with VIF ≥ 10 sequentially). An “Embedded” algorithm was applied to winnow markers based on their slopes (regarded as their importance) in the model sequentially until optimal performance was achieved. L2 regulation was implemented to improve robustness of the model.

Secondly, in the random forest model, hyperparameters (e.g., number of estimators, maximum depth, minimum sample leaf, maximum features, and minimum sample split) were self-optimized through grid search. Variable importance was evaluated by the Gini importance (GIM) as in Equations (1) and (2):

(1) $$GIM=\sum_{i=1}^{I}\left({GI}_{q}^{i}-{GI}_{l}^{i}-{GI}_{r}^{i}\right)$$

(2) $${GI}_{q}^{i}=1-\sum_{c=1}^{\left|C\right|}{\left(p_{qc}^{i}\right)}^{2}$$

In which “*i*” denotes a single decision tree; “*I*” signifies the ensemble of trees; “*q*” represents the father leaf while “*l*” and ‘*r*’ represent the left and right child leaves, respectively; “*c*” indicates the category *c* in ensemble “|*C*|”; “*p_qc_*” signifies the proportion of category *c* in the leaf *q*.

Thirdly, in SVM models, kernel functions (linear, sigmoid, and gaussian radial basis function kernel) were applied. Similarly, hyperparameters (regularization parameter, degree, and gamma) were optimized using the grid searching method. The importance of a predictor in a linear kernel model was reported by the coefficient of the support vector.

**Figure 1:**
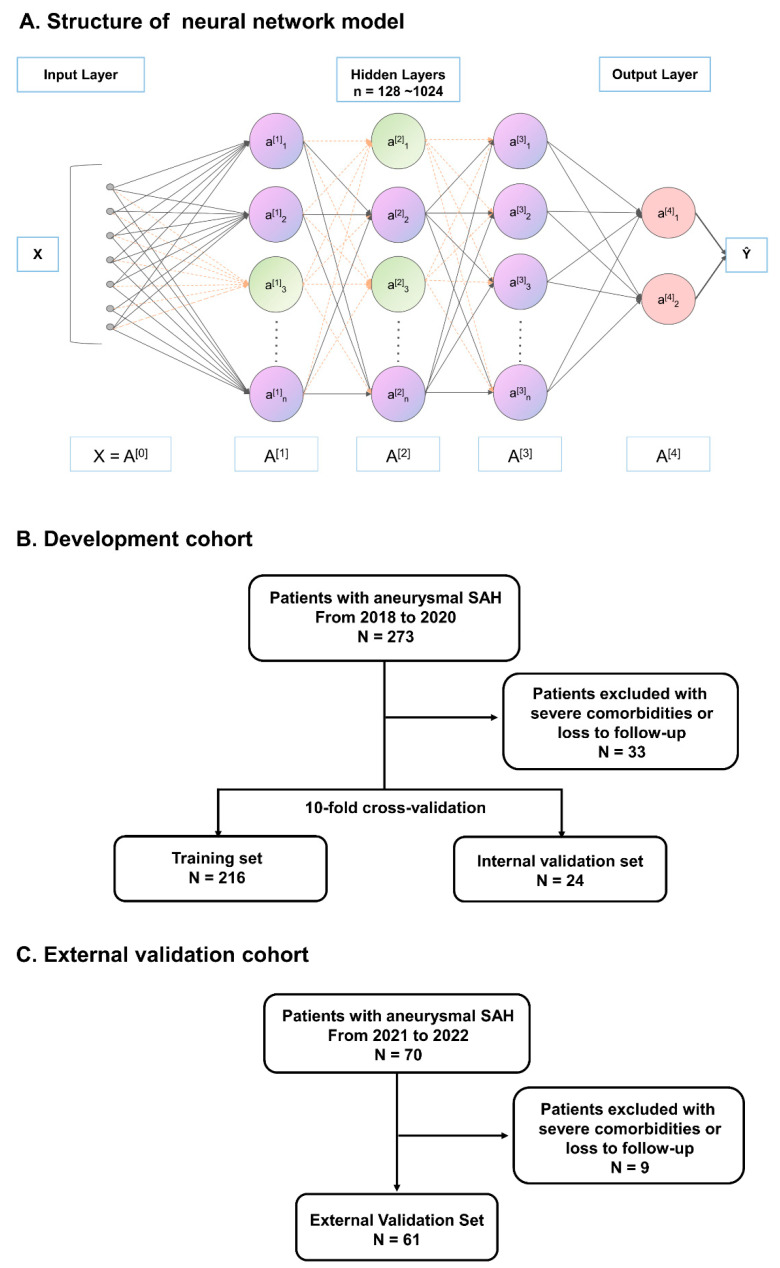
Overview of the neural network and flowchart of patient selection. (**A**) A FCNNs model with 3 hidden layers and varying numbers of neurons (128, 256, 512, or 1024). Linear transformations, ReLU activations, dropout (green neurons), and sigmoid/softmax outputs were adopted to automatically learn nonlinear relationships from data for prediction. (**B**) Cohort for model development. (**C**) External validation cohort for scorecard model.

Fourthly, the FCNN model is shown in Figure 1A. Briefly, in view of the data type, FCNN was adopted with one input layer, three hidden layers, and one output layer. Four types of structures, each with three hidden layers, containing 128, 256, 512, or 1024 neurons, were adopted. The input vector from the former layer was linearly transformed through a specific weight matrix. The Rectified Linear Unit (ReLU) was then applied as the activation function. To reduce the overfitting, random dropout was carried out in the first two hidden layers. In the output layer, predictions were generated after “sigmoid” and “softmax” function. The process was completed using PyTorch (Version 1.13.0) and PyTorch Lighting (https://lightning.ai/docs/pytorch/latest/, accessed on 18 May 2023) (Version 1.9.4) in Python (Version 3.7.4). Model performance was assessed by accuracy and area under the ROC curve (AUC) with 95% confidence intervals (CIs) after 10-fold cross-validation. All processes were performed using the Scikit-learn package (Version 0.24.2) in Python (Version 3.9.7).

Given that machine learning algorithms may encounter issues with simplicity and interpretability, a scorecard model was developed based on credit risk scorecards used in the financial industry [18]. In this model, quantile binning was firstly adopted for each predictor. The number of bins was 20 for continuous variables and adjusted for discrete variables. The weight of evidence (WOE), an index defining the relationship between the potential predictor and the binary target label, was calculated for each bin as in Equation (3). Subsequently, the information value (IV), an indicator assessing the strength of that relationship, was computed for each predictor, as in Equation (4).

(3) $${WOE}_{i}=\ln\left(\frac{{Bad}_{i}}{{Bad}_{T}}\right)-\ln\left(\frac{{Good}_{i}}{{Good}_{T}}\right)$$

(4) $$IV=\sum_{i=1}^{N}\left(\left(\frac{{Bad}_{i}}{{Bad}_{T}}-\frac{{Good}_{i}}{{Good}_{T}}\right)*{WOE}_{i}\right)$$

In Equations (3) and (4), “*WOE_i_*” denotes the WOE of a divided bin. “*Bad_i_*” and “*Good_i_*” represent the patient’s number of bad outcomes and good outcomes in this bin, while “*Bad_T_*” and “*Good_T_*” represent the total number of patients with bad and good outcomes, respectively. These two indices evaluate valuable importance and dividing lines through distribution differences based on relative entropy. For feasibility and simplicity, the chi-square between adjacent bins was calculated, and the two bins with the highest chi-square were merged until only two or three bins remained in each variable. Predictors with VIF < 10 and IV ≥ 0.1 with two or three bins were selected as the target ones. The score of every remaining bin was calculated as in Equation (5), where the odds ratio is the ratio of the probability of a good outcome to the probability of a bad outcome. To constrain the final score distribution, the scoring system was calibrated such that an odds ratio increase from the initial data ratio to 95%/5% corresponded to a score change of 50 units. Logistic regression model performance was used to measure our scorecard, and the number of bins (2 or 3) for each predictor was decided based on the best performance. Another external validation cohort was collected to reassess its performance.

(5) $$Score=Offset-Scale*\log\left(odd\right)$$

## 3. Results

### 3.1. Patient Data Collection and Demographics

A total of 273 aSAH patients were initially enrolled in the study. However, 25 patients were lost to follow-up (9.16%), and 8 had comorbidities such as malignant tumors or severe hepatic and renal dysfunction (2.93%). Finally, 240 patients were included in the cohort for model development (Figure 1B). In the external validation cohort for the scorecard model, of the 70 patients, 9 (12.86%) were excluded from the study based on the aforementioned exclusion criteria (Figure 1C).

Table 1 shows the comparable baseline characteristics between groups (mean age: 56 vs. 55 years; female: 62.5% vs. 60.66%), indicating generalizability. The aneurysms were most frequently located in the anterior circulation and were clipped in over 60% of patients. While minor differences in aneurysm locations, clinical grading scales, and radiology scores were observed between aSAH cohorts, measures of clinical severity and blood cells on admission were generally consistent.

### 3.2. Prediction Models

**Figure 2:**
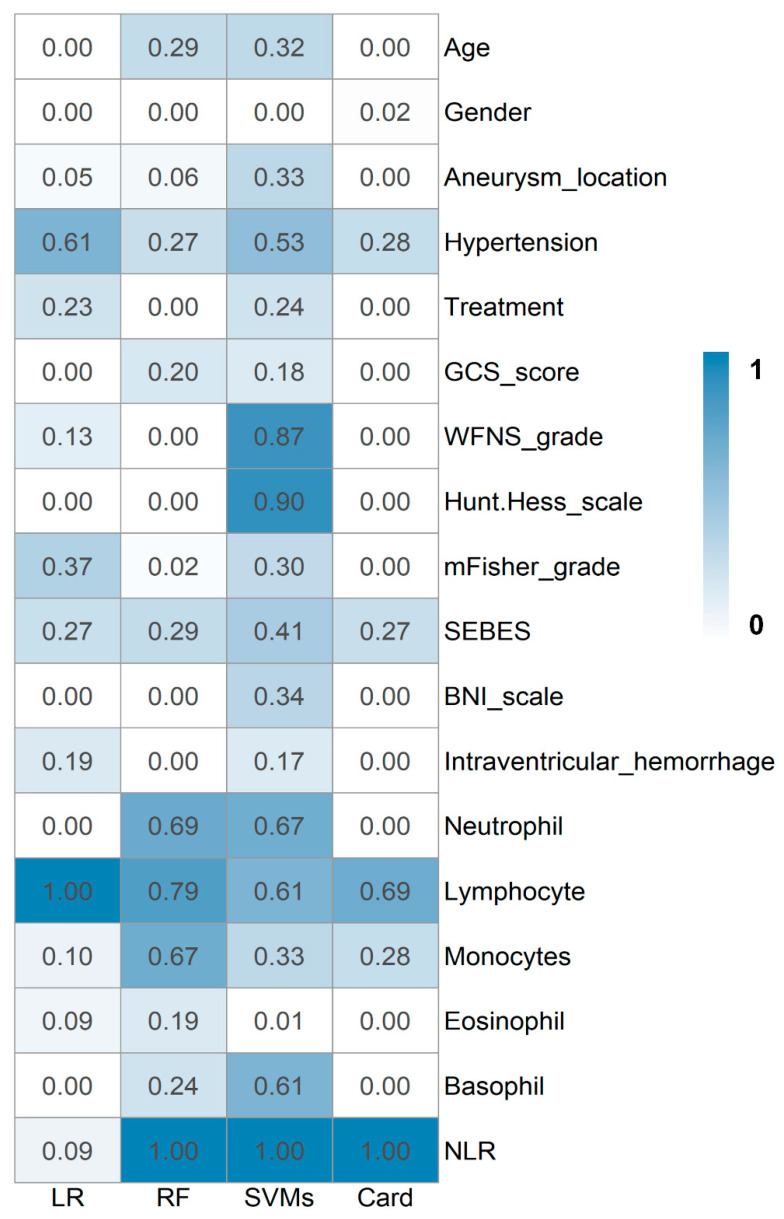
Relative importance of variables for models. LR, logistic regression model; RF, random forest model; SVMs, support vector machine models; Card, scorecard model; GCS_score, Glasgow Coma Scale; WFNS_grade, World Federation of Neurological Surgeons grade; SEBES, Subarachnoid Hemorrhage Early Brain Edema Score; BNI_scale grade: Barrow Neurological Institute scale; NLR, the neutrophil-to-lymphocyte ratio.

In the logistic regression model, 11 variables were ultimately selected after self-optimization: aneurysm location, hypertension, treatment, WFNS grade, mFisher grade, SEBES, intraventricular hemorrhage, lymphocyte count, monocyte/macrophage count, eosinophil count, and NLR (Figure 2). Among the variables in the model, lymphocyte count carried the highest weighting, followed by hypertension, with the second-highest weighting, and modified Fisher grade, with the third-highest weighting. The logistic regression model had an optimal accuracy of 0.792 (0.745–0.828) and an AUC of 0.739 (0.645–0.834) (Table 2).

Random forest is an ensemble learning method for classification, regression, and other tasks. It operates by constructing multiple decision trees during training and outputs the class that is the mode of the classes (for classification) or the mean prediction (for regression) of the individual trees, thereby correcting for decision trees’ tendency to overfit to their training set. In our study, a random forest with 401 trees was created after training (maximum depth: 1; minimum sample leaf: 1). Twelve predictive factors were selected to achieve optimal performance with an accuracy of 0.783 (0.770–0.786) and an AUC of 0.749 (0.664–0.834) (Table 2). In this model, the top six important variables were NLR, lymphocyte count, neutrophil count, monocyte count, age, and SEBES (Figure 2).

SVMs are a set of supervised learning methods. They identify the optimal boundary between classes by maximizing the margin, which is the distance between the separating boundary and the data points (support vectors) closest to it. Using a traditional linear kernel function, the top four important factors were found to be NLR, Hunt and Hess scale, WFNS grade, and neutrophil count (Table 2). The accuracy was 0.796 (0.742–0.849), and the AUC was 0.762 (0.679–0.844). Kernel functions are a key concept in SVMs, enabling them to classify nonlinearly separable data by transforming the data from the original input space into a higher dimensional space. They help SVMs identify optimally separating hyperplanes in high-dimensional feature spaces without requiring explicit transformation of the original data. Here, two nonlinear kernel functions were used (Gaussian radial basis function and sigmoid kernel function), with reported accuracy (0.783, 0.771–0.796 vs. 0.821, 0.766–0.875) and AUC (0.774, 0.696–0.852 vs. 0.793, 0.723–0.863), respectively (Table 2).

FCNNs contain multiple hidden layers of neurons between the input and output layers. The hidden layers enable the network to learn complex patterns in the data, and four numbers of neurons in the hidden layers were adopted (128, 256, 512, and 1024). Among the numerous neurons, a regularization technique called random dropout was applied, where randomly selected neurons are ignored during training to help prevent overfitting by dropping out correlated neurons in each iteration. The performance of the four FCNNs models is illustrated in Table 2, with both accuracy and AUC approximating 0.95.

### 3.3. Scorecard Model

Despite enhancing predictive capabilities, machine learning algorithms can pose challenges regarding model interpretability and clinical utility. Therefore, to complement the machine learning approaches, a straightforward clinical scorecard model was developed to enable prediction with improved transparency and ease of use for practitioners. In this study, information value, calculated through entropy reduction, was employed to quantify the predictive capacity of attributes. An attribute with high discriminative power to reliably discern group membership will produce a substantial decrease in entropy and thus exhibit elevated information value. Thus, after eliminating multicollinearity, five final predictive factors were screened: NLR, lymphocyte count, monocyte count, hypertension, and SEBES (Figure 2). According to their weights of predictive effect, the cutoff values and relative scores were determined by the algorithm (Table 3).

The cutoff values of NLR, lymphocyte count, monocyte count, and SEBES were 8.301 and 17.233, 0.68 and 1.523 × 10^9^/L, 0.179 × 10^9^/L, and 2.0, respectively. After assigning the corresponding scores, the performance of the scorecard was firstly evaluated by the internal validation set, with a reported accuracy of 0.813 and an AUC of 0.794. Meanwhile, in the ROC curve, a total score of five was determined as the optimal cutoff value for distinguishing favorable and unfavorable outcomes. To more accurately evaluate the robustness and reliability of the established scorecard, an additional external validation cohort of 61 patients was collected, in which the accuracy of the scorecard was 0.787.

## 4. Discussion

In this study, to predict outcomes of patients with aSAH using admission information, 18 easily available variables were initially collected, including medical history, admission status, neuroradiological data, and blood routine examination results. Using the training and internal validation sets, the performance of LR, RF, SVM, and FCNNs models was compared. The accuracy of the traditional LR model was higher than that of the RF model (0.792 vs. 0.783), whereas the AUC was lower (0.739 vs. 0.749). For the SVMs, both the accuracy and AUC were slightly higher than those of the LR model, and an impressive improvement was observed in the FCNNs model (up to nearly 0.95). These data verified the predictive power of machine learning algorithms.

The key principle of LR is a linear transformation of the input variables. In medicine, with increasing numbers of variables and participants, the amount of data may not reveal all the features through a simple linear transformation [19]. RF is an ensemble of decision trees that aims to maximize information gain or decrease entropy (the uncertainty or impurity in the data). It is a nonlinear modeling technique that partitions the input space into rectangular regions nonparametrically [20]. Kernel functions, which are integral to SVMs, implicitly facilitate the classification of nonlinearly separable data by transforming the data from the original input space into a higher dimensional space. As a result, optimal separating hyperplanes can be identified in the higher dimensional space by SVMs [21]. In our study, while the kernel functions were switched from the linear kernel to the Gaussian radial basis and sigmoid kernels, the nonlinear portion increased; thus, the AUC improved, although the accuracy fluctuated. FCNNs are a type of artificial neural network that use nonlinear transformations to model complex relationships, where hidden layers learn hierarchical representations of the data by combining the features detected by lower layers into more abstract concepts [22]. Four numbers of neurons in the hidden layers were evaluated, and all performed exceptionally well. As the number of neurons in each hidden layer increased (128 to 256, 512, and 1024), the accuracy improved, although the AUC changed slightly. Meanwhile, the mean number of epochs taken to reach convergence decreased (125.4 to 90.9, 84.6, and 60.5, respectively), indicating an increasing speed of the FCNNs to learn these hierarchical representations. As for the scorecard model, although the key concept in training is still the linear method of logistic regression, the process of rating and scoring is technically a nonlinear transformation to separate data according to their distributions. This may result in a better performance of the scorecard model compared to the logistic regression model (accuracy: 0.813 vs. 0.792; AUC: 0.794 vs. 0.739) using only 5 variables versus 11 predictors in the logistic regression model.

The rupture of an intracranial aneurysm leads to bleeding into the subarachnoid space, inducing complex cerebral, systemic, and microvascular reactions including acute transient global ischemia, blood–brain barrier disruption, inflammation, cortical spreading depolarizations, and multi-organ dysfunction [23,24]. Our results demonstrate that the neutrophil-to-lymphocyte ratio (NLR) is one of the most significant predictors of outcome across our multiple models. This stems from two NLR components: excessive neuroinflammation indicated by neutrophilia, and immunosuppression indicated by lymphocytopenia [25]. Firstly, SAH induces an inflammatory cascade marked by neutrophil mobilization, release of acute-phase proteins like the C-reactive protein and cytokines, including interleukin-6. Concurrently, hemorrhage disrupts the blood-brain barrier, enabling inflammatory mediators and cells to penetrate brain tissue. Neutrophils adhere to the cerebrovascular endothelium within minutes post-hemorrhage, then infiltrate the injured brain parenchyma in response to chemokines [26,27,28]. While needed for clearing erythrocyte debris initially, excess neutrophil activation and infiltration cause damage via enzymes, radicals, and neutrophil extracellular traps [29]. Secondly, post-SAH lymphocytopenia denotes immunosuppression, potentially from redistribution and apoptosis [30,31]. This predisposes patients to infections like pneumonia, ventriculitis, and urinary tract infections. Infections, especially in elderly patients, can prolong hospitalization and hinder recovery, contributing to poorer 3-month outcomes. Lymphopenia may also directly impair neuroprotective, heme-clearing, and tissue repair functions [32,33]. As a result of these effects, higher NLR tended to present with more severe clinical conditions (e.g., coma, heightened inflammatory state, neurological dysfunctions), suggesting bad WFNS, GCS, and Fisher score, indicating poorer outcomes [11,31,34].

Monocytes are important innate immune cells that regulate inflammation through cytokine production and antigen presentation. After SAH, traditionally, it was thought that mobilizing and trafficking monocytes infiltrated the brain, differentiating into macrophages that likely contributed to secondary injury through promoting inflammation. However, evidence suggests that these cells can also facilitate tissue repair, beneficial immunomodulation, and debris clearance in later phases [35,36]. Our data also illustrated the relevance of the monocyte count. Additionally, the impact of hypertension to SAH is extensive, relating to the pathogenesis, risk factor, exacerbation, and complications from the pathophysiological effects, amplifying the acute cerebrovascular dysfunction after SAH [37]. It leads to vascular remodeling and impaired autoregulation, increasing susceptibility to ischemic injury after hemorrhage. Hypertension also damages blood–brain barrier integrity, exacerbating vasogenic edema and neurotoxicity. Uncontrolled high blood pressure impedes cerebral perfusion, compounding the reduction in cerebral blood flow after SAH, thus indicating a poor outcome [38,39]. Early Brain Edema Score (SEBES) emerges as another consistent predictor, which is a semiquantitative CT grading scale ranging from 0 to 4. It indicates the edematous change through the absence of visible sulci caused by the effacement of sulci or disruption of the gray–white matter junction [13]. The SEBES can reflect the severity of early vasogenic and cytotoxic edema. After aneurysm rupture, blood–-brain barrier disruption and cellular swelling worsen edema [40]. The resulting intracranial pressure elevation and delayed cerebral ischemia may severely impact outcomes [41,42]. In summary, our data implicate inflammatory responses, monocyte infiltration, hypertension, and cerebral edema as key factors determining SAH outcome.

While the present study included 301 participants, the sample size is relatively modest compared to large-scale research involving thousands of subjects. The single-center design introduces potential systematic bias, which is common in smaller studies. The imbalanced distribution (e.g., treatment and GCS) of the data, with fewer examples of the minority class, poses inherent risks of overfitting on the majority class and underfitting on the minority class when using machine learning algorithms. Thus, the generalization of the model should be applied with caution. To provide a more comprehensive evaluation of model performance, we reported both accuracy and AUC based on precision and recall. However, residual selection bias from the original imbalanced dataset may still lead to reduced model generalizability and stability. The modest sample size likely constrained our ability to detect smaller effect sizes or subgroup differences. Multi-center studies with larger, more balanced samples will be needed to validate and extend the current findings. We aim to conduct such research in the future to further advance predictive modeling in this application domain.

## 5. Conclusions

This study demonstrated that machine learning algorithms could enhance the prediction efficacy for outcomes in individuals with aSAH using medical history, admission status, neuroradiological data, and blood analysis results. Among these potential predictors, NLR, lymphocyte count, monocyte count, hypertension status, and SEBES may be relatively important. Through nonlinear transformation, a simplified scorecard system was established to predict outcomes using only five factors for clinicians’ convenience.

## Figures and Tables

**Table 1:** Demographics of patients. For each predictor, mean ± SD, median (IQR), and quantity (proportion) are shown for continuous variables, discrete variables, and binary variable (yes/no), respectively.

Predictors	Development Cohort (*n* = 240)	Validation Cohort (*n* = 61)
Age (y)	56.18 ± 11.69	54.77 ± 12.47
Female	150 (62.5%)	37 (60.66%)
Aneurysm location		
Anterior cerebral artery	102 (42.5%)	24 (39.34%)
Middle cerebral artery	39 (16.25%)	17 (27.87%)
Posterior circulation	84 (35%)	12 (19.67%)
Internal carotid artery	15 (6.25%)	8 (13.11%)
Hypertension	112 (46.67%)	26 (42.62%)
Treatment		
Coil	91 (37.92%)	21 (34.43%)
Clip	148 (61.66%)	40 (65.57%)
Conservative treatment	1 (0.42%)	0 (0%)
GCS score		
3–8	33 (13.75%)	10 (16.39%)
9–12	6 (2.5%)	3 (4.92%)
13–15	201 (83.75%)	48 (78.69%)
WFNS grade		
1	174 (72.5%)	42 (68.85%)
2	24 (10%)	5 (8.20%)
3	3 (1.25%)	1 (1.64%)
4	24 (10%)	4 (6.56%)
5	15 (6.25%)	9 (14.75%)
Hunt and Hess scale		
1	81 (33.75%)	6 (9.84%)
2	83 (34.58%)	34 (55.74%)
3	46 (19.17%)	11 (18.03%)
4	26 (10.83%)	7 (11.48%)
5	4 (1.67%)	3 (4.92%)
Neuroimaging assessment		
mFisher grade	3 (2–4)	3 (2–4)
SEBES	1 (0–2)	2 (0–3)
BNI scale	5 (4–5)	5 (4–5)
Intraventricular hemorrhage	132 (55%)	41 (67.21%)
Laboratory indexes		
Neutrophil (×10^9^/L)	11.05 ± 3.95	12.55 ± 5.14
Lymphocyte (×10^9^/L)	1.10 ± 0.54	1.02 ± 0.43
Monocytes (×10^9^/L)	0.52 ± 0.33	0.59 ± 0.36
Eosinophil (×10^9^/L)	0.01 ± 0.03	0.01 ± 0.02
Basophil (×10^9^/L)	0.03 ± 0.02	0.02 ± 0.01
NLR	11.77 ± 5.85	14.45 ± 8.43

**Table 2:** Performance of predictive models. Accuracy and AUC were reported with 95% CI. Three kernel functions (linear, sigmoid, and gaussian radial basis function kernel) were applied in SVM models. Four types of structures with 128, 256, 512, or 1024 neurons in hidden layers were adopted in FCNNs model. rbf, gaussian radial basis function; FCNNs, fully connected neural networks.

Models		Accuracy	AUC
Logistic Regression		0.792 (0.745–0.828)	0.739 (0.645–0.834)
Random Forest		0.783 (0.770–0.786)	0.749 (0.664–0.834)
Support Vector Machine	linear	0.796 (0.742–0.849)	0.762 (0.679–0.844)
	rbf	0.783 (0.771–0.796)	0.774 (0.696–0.852)
	sigmoid	0.821 (0.766–0.875)	0.793 (0.723–0.863)
FCNNs	128	0.950 (0.927–0.972)	0.946 (0.918–0.974)
	256	0.957 (0.938–0.976)	0.952 (0.920–0.983)
	512	0.960 (0.940–0.979)	0.946 (0.911–0.981)
	1024	0.962 (0.947–0.978)	0.947 (0.916–0.978)

**Table 3:** Scorecard system for predicting outcomes of patients with aSAH. NLR, the neutrophil-to-lymphocyte ratio; SEBES, Subarachnoid Hemorrhage Early Brain Edema Score; inf, infinite.

Variables	Range	Score
NLR	(−inf, 8.301]	−14
	(8.301, 17.233]	−1
	(17.233, inf)	15
Lymphocyte count	(−inf, 0.68]	7
	(0.68, 1.523]	−1
	(1.523, inf)	−8
Monocyte count	(−inf, 0.179]	12
	(0.179, inf)	−1
Hypertension	No	−5
	Yes	4
SEBES	(−inf, 2.0]	−3
	(2.0, inf)	9

Total score > 5 indicates poor outcome.

## Data Availability

The data presented in this study are available from the corresponding author upon reasonable request.

## Abbreviations

aSAH	aneurysmal subarachnoid hemorrhage
BNI	Barrow neurological institute
FCNN	fully connected neural networks
GCS	Glasgow Coma Scale
IV	information value
LR	logistic regression model
NLR	the neutrophil-to-lymphocyte ratio
RF	random forest model
SEBES	subarachnoid hemorrhage early brain edema score
SVM	support vector machine
WFNS	World Federation of Neurological Surgeons
VIF	variance inflation factor
